# The Great Meeting at Barts

**Published:** 1888-11-24

**Authors:** 


### THE GREAT MEETING AT BARTS.

BY ONE WHO WAS THERE.

A GOOD deal of significance attaches to the meeting at St. Bartholomew's Hospital on the 15th inst. Sir Sydney Waterlow gave the results of his sixteen years' experience as Chairman of the Distribution Committee of the Hospital Sunday Fund. The new Lord Mayor, Mr. Alderman Whitehead, as President of the Fund, occupied the chair. No one who knows Sir Sydney Waterlow will call him a man of extremes. He is a typical Englishman of the kindly, common-sense type. He wishes everybody to be helped who needs help, and he desires that the kindness of the helpers shall not be abused. It need not be doubted that Sir Sydney knows the value of money. No one could have built up the colossal business of which he is the head who did not know that. Not only does he know the value of his own money, but also that of other people's. This is one of his strong points, as it is one of the weak points of so many hospital managers. The Hospital Sunday Fund amounts to nearly £40,000 every year—a large sum ; and Sir Sydney Waterlow, as the superintendent of its distribution, feels his responsibility very deeply.

But this sense of responsibility is the very thing that is most wanted in all public men. Especially is it wanted in those who have charge of public money. What does public money represent? The brains, the labour, the anxiety, the mind and heart, the flesh and blood of the people. Earned money stands over against earning power as its complete equivalent; and earning power in these times of pressure means almost all the power men and women possess. For he who does not use his most strenuous exertions is in danger, not only of being left behind in the race, but of being thrown into the hedge or gutter by the wayside, like the dirty rags despised by the beggar. It is a great thing to have a clear comprehension of the value of money; it is an honest and worthy thing to have as clear a comprehension of the value of other people's as of one's own. This worthy elevation Sir Sydney Waterlow has reached.

The keynote of the paper read by Sir Sydney on Thursday evening was the avoidance of waste and extravagance. There ran through the whole speech an undercurrent of fear of small and special hospitals, and a firm note of confidence in large general hospitals. No charge was made or even hinted against the management of special hospitals. Costliness is inherent in the system itself of many hospitals, as against few. There is no mystery or difficulty in this. For the proper working of a hospital a certain "plant," to use a business term, is necessary, and certain officials are indispensable. This "plant" and these officials are required whether the hospital is large or small. The "plant" and the officials entail a large proportion of the cost of the whole administration. The "plant" and the officials which are sufficient for a hospital of twenty beds are almost sufficient for one of fifty. The "plant" and officials which are needed for a hospital of a hundred beds are almost sufficient for one of three hundred. It is plain, then, that the indefinite multiplication of small and special hospitals means the indefinite multiplication of separate "plants" and separate sets of officials, and a consequent increase of cost which is enormous. The business instincts of an experienced man like Sir Sydney Waterlow show him that this is not as it should be. On no consideration would he carry on his own large business in any such extravagant way.

The meeting as a whole undoubtedly went with the speaker. Mr. Lennox Browne entered an urgent plea in favour of special hospitals, and everyone gladly admitted that many years ago some of these institutions had rendered excellent service; that still there are degrees of merit in special hospitals; that some have shown a fitness for survival, whilst others stand self-condemned and only await the slow decomposition of time. But notwithstanding Mr. Lennox Browne's able advocacy, common sense, and business instincts prevailed, and Sir Sydney's contention in favour of the affiliation of most of the special hospitals to the large general hospitals had the best of the running. Mr. Burdett, who has studied the question more than any other man, was decidedly in favour of the less costly method of affiliation, as also, we believe, was Dr. Matthews Duncan, although he did not publicly express his conviction. Dr. Steele and other speakers followed on the same lines. Mr. Lennox Browne had perhaps one sympathiser in Mr. Power, who spoke in the kindest and most genial manner, and produced a favourable impression. If able advocacy could have carried the day, the special hospitals would have had a chance of a more favourable verdict. But advocacy notwithstanding, the net result of the meeting went to show that the multiplication of special hospitals is not a desirable thing, and that, as a rule, it would be a saving of money and an increasing of efficiency in all directions if most of the present special institutions could be affiliated to large general hospitals.

A decisive word was said by Dr. G. W. Potter in favour of a better distribution of the metropolitan medical charities. It is not generally known that nearly all the large, and even the small hospitals, are situated within a radius of a mile and a-half from Charing Cross. From this it results that that portion of London has too many hospitals by half, whilst many outlying districts, where poor persons, who most need hospitals, live in tens of thousands, have no hospital accommodation at all. The north of London, the extreme east, and the extreme south are practically destitute of hospitals. If it be asked why these institutions are all so crowded together in mid-London, the answer is that they are there for the convenience of the physicians and surgeons. If again it be asked if there are no physicians and surgeons in the suburbs capable of treating hospital cases efficiently, the answer again is "hundreds"; but the present system of making appointments to hospitals excludes the whole profession, except a mere handful of men whose special claim to consideration is as shadowy and impalpable as the ghost of Hamlet's father. The question amounts to this: Should hospitals be placed among the poor and the sick where they are needed, or should they be placed miles away from them? There was a time when the City was full of churches, as indeed it is now; but although the City churches are still standing, every suburb has its score of new and handsome buildings for the supply of spiritual needs. If the churches have followed the population, should not the hospitals follow it much more?

The Hospitals Association, under whose auspices this most successful meeting was held, is to be congratulated upon its growing popularity and its rapidly-increasing usefulness. St. Bartholomew's Hospital, whose large and majestic hall was given for the occasion, also deserves well of the hospital world for its practical sympathy with reformed hospital administration. The hall presented a picturesque and unique appearance. There were many distinguished ladies and gentlemen present; but they were nowhere compared with the long rows of white-capped, white-aproned, spotlessly clean, and most neat and gentle-looking nurses. If any impressionable bachelors were present they could not but have thought in their hearts how desirable it would be to choose a wife from among those who have had the admirable training in practical woman's work which every hospital nurse has to pass through. The meeting was most successful. As the French say, "All the world was present, and all the world was pleased."

